# Evaluation of the non-auditory neurocognitive test MoCA-HI for hearing-impaired

**DOI:** 10.3389/fneur.2022.1022292

**Published:** 2022-12-13

**Authors:** Christiane Völter, Hannah Fricke, Lisa Götze, Franziska Labrenz, Marianne Tokic, Rainer Wirth, Ziad S. Nasreddine, Piers Dawes

**Affiliations:** ^1^Department of Otorhinolaryngology, Head and Neck Surgery, Catholic Hospital Bochum, Bochum, Germany; ^2^Department of Medical Psychology and Medical Sociology, Faculty of Medicine, Ruhr-University Bochum, Bochum, Germany; ^3^Department of Medical Informatics, Biometry and Epidemiology, Ruhr-University Bochum, Bochum, Germany; ^4^Department of Geriatric Medicine, Marien Hospital Herne, Ruhr-University Bochum, Herne, Germany; ^5^MoCA Clinic and Institute, Greenfield Park, QC, Canada; ^6^School of Health and Rehabilitation Sciences, University of Queensland, Brisbane, QLD, Australia

**Keywords:** hearing loss, cognitive screening, MoCA, test battery, dementia

## Abstract

**Background:**

Since hearing loss and cognitive decline often co-occur among older adults, a cognitive screening test suitable for hearing-impaired people is of high clinical relevance. We report the first evaluation of a German language version of the Montreal Cognitive Assessment—Hearing Impaired version (MoCA-HI).

**Objective:**

The aim of the present study was to compare cognitively healthy participants with and without hearing loss, to examine the impact of age, sex, educational level and degree of hearing impairment on the German MoCA-HI performance, and to develop normative data.

**Material and methods:**

The German MoCA-HI was tested in 94 participants with normal or mild hearing impairment (group 1: 4PTA ≤ 40 dB on the better hearing ear) and 81 participants with moderate to profound hearing loss (group 2: 4PTA > 40 dB on the better hearing ear). Additionally, all participants performed the standard MoCA (version 8.2).

**Results:**

No significant group difference between group 1 and 2 was found in the MoCA-HI total score (*p* = 0.05). In contrast, group 1 performed significantly better than group 2 on the standard MoCA (*p* < 0.001). There was no difference between the MoCA and the MoCA-HI performance in group 1 (*p* = 0.12), whereas individuals of group 2 performed significantly better on the MoCA-HI than on the standard MoCA (*p* < 0.001). Test-retest reliability of the MoCA-HI was high (*p* < 0.001). Higher age (*p* < 0.001), male sex (*p* = 0.009) and lower education (*p* < 0.001) were associated with a lower overall MoCA-HI score. Based on the demographic data normative data were developed by a regression-based approach.

**Conclusion:**

The MoCA-HI is a cognitive screening test which is suitable for people with hearing impairment.

## Introduction

Age-related hearing loss and dementia are among the most common chronic diseases in old age. Currently, approximately 430 million people live with disabling hearing loss (1), while 55 million people worldwide have dementia (2). Hearing loss and dementia are commonly co-morbid. Age-related hearing loss is associated with increased risk for cognitive impairment, increasing likelihood of comorbidity of hearing loss with cognitive impairment (3–7). One survey of people with cognitive impairment attending a memory clinic reported that around 85% had a hearing impairment (8).

There is a growing interest in neurocognitive testing in settings outside psychologic or psychiatric ones (9, 10), particularly in hearing rehabilitation settings. Routine hearing assessments depend on cognitive function; tests of speech recognition, for example, are impacted by cognitive factors (11). A patient's cognitive profile is increasingly taken into account in auditory rehabilitation in cochlear implant patients (9, 12–14) and speech recognition outcomes among cochlear implant recipients are better for those with better cognitive ability (15, 16).

Numerous screening tests are available to identify cases of cognitive impairment (17). However, these tests mostly involve spoken stimuli, and persons with hearing loss (or under conditions of simulated hearing loss) perform worse than those with normal hearing (18–20). Hearing impairment may lead to false-positive diagnosis of dementia and/or overestimation of cognitive impairment (19).

Several attempts have been made to adapt cognitive screening tests for people with hearing loss (21, 22). Adaptations included deleting spoken items or presenting spoken items in visual format. Although these adaptations can impact the psychometric properties of the tests [e.g., (23)], the sensitivity and specificity of the adapted versions have mostly not been established.

Dawes et al. developed a visual version of the Montreal Cognitive Assessment (MoCA) (24) for people with hearing impairment (25) and validated it in 461 participants with combinations of hearing and cognitive impairment. It has shown a good sensitivity and specificity for the detection of dementia of 95.74 and 85.71% respectively, at a cut-point of ≤ 24 points with a 2-point adjustment for education, comparable to the standard MoCA (www.mocatest.org). This MoCA-HI is freely available from the MoCA website (www.mocatest.org) to appropriately trained persons after a short fee-based online training offered by the same website. In Dawes et al.'s version, the spoken items of the standard MoCA (version 8.1) were presented visually (e.g., with written instructions) or substituted with alternative visual tasks (e.g., the sentence repetition task was replaced by a sentence formation task). These adaptations were designed to index the same cognitive domain as the standard items and to be of a similar level of difficulty.

Dawes et al.'s validation was carried out using an English version of the MoCA-HI (26). Pooling data across different languages for analysis is planned as differences in performance between different language translations have been reported for the original MoCA and may be due to cultural or linguistic impacts or differences in dementia diagnosis between countries (27). An implication is that performance criteria to identify cognitive impairment derived in English may not be applicable to other languages. Translated versions of the MoCA should be re-validated with local populations. Therefore, we developed a German language translation of the MoCA-HI (28).

The aim of the present study was (1) to compare performance of the German version of the MoCA-HI and the original MoCA in cognitively healthy participants with and without hearing loss, (2) to examine the impact of age, education, sex and level of hearing loss on performance and (3) to derive corresponding performance norms of the German MoCA-HI.

## Materials and methods

Inclusion criteria were as follows: (1) age of 60 years or older, (2) native or excellent German speaker, (3) normal or corrected near visual acuity of ≤ 0.3 logMAR, (4) normal performance in the GPCOG (General Practitioner Assessment of Cognition) (29) (a score of 9 points) or a GPCOG score between 5-8 in combination with the additional informant questionnaire of the GPCOG with a score of 4–6 points, (5) GDS-15 (Geriatric Depression Scale - 15) in the normal range (30). Participants with a cognitive impairment as shown by the GPCOG or by medical history, and those with a severe neurological or psychiatric disease or a severe motor disorder that might interfere with testing were excluded. Pure tone audiograms at 500, 1000, 2000 and 4000 Hz for each ear separately were performed with headphones, and visual acuity was examined using a near vision panel. Based on the hearing thresholds, participants were divided into two groups according to the WHO classification (31). Group 1 included normal/mild hearing-impaired participants (4PTA on the better hearing ear ≤ 40 dB), which refers to WHO grade 0 and 1 and group 2 included the moderate to profound hearing loss group (4PTA on the better hearing ear > 40 dB), which refers to WHO grade 2, 3 and 4. MoCA and GPCOG testing were done with hearing devices, MoCA-HI testing without a hearing aid or a cochlear implant. All participants performed the MoCA-HI (Version 1.0 German) and the two spoken tasks of the standard MoCA (Version 8.2), i.e., the list of letters and the sentence repetition. A retest of the MoCA-HI was conducted in 115 participants after at least 4 weeks.

### Statistical analysis

To achieve a medium effect size for a group comparison using a *t*-test at an alpha level of 0.05 with a power (1-beta) of 0.90, two groups of at least 70 participants were required. In total, 175 participants were included (group 1: *n* = 94; group 2: *n* = 81). Descriptive statistics including mean (*M*) and standard deviation (*SD*) were used to describe sociodemographic, audiological and cognitive data. *T*-tests were performed to compare group 1 to group 2 with regard to age, education, MoCA-HI total-score and the individual cognitive subdomains and reported by mean difference (*MD*) and *p*-value. To compare the results of the two groups in the adapted tasks of the MoCA-HI and the corresponding tasks of the standard MoCA, the Mann-Whitney-U-test was used. To examine performance differences between the MoCA and the MoCA-HI within each group, the Wilcoxon signed rank test was applied. In order to analyze the impact of hearing impairment on the MoCA-HI-total-score and the cognitive subdomains, multiple regression analysis was carried out including the 4PTA as a continuous measure of hearing ability taking into account age, education and sex. Test-retest-reliability was determined by a Pearson-correlation of the MoCA-HI total-scores at both measurement time points.

Normative scores of the MoCA-HI, taking into account age, education, and sex, were developed for the age group from 60 to 97 years. A regression-based approach which allows to account for multiple variables and analyzes continuous variables such as age and education across the entire range, was chosen (32–35). The uncorrected MoCA-HI total score (without the 2 points for ≤ 12 years of education) was used (35). First, 20 different general linear models were examined, as described by (36). For this purpose, 5 basic regression models, the squared covariates and their interaction with sex were tested. The best model was defined as the one that had the minimum predicted residual sum of squares (PRESS) statistic with PRESS = $\sum {\left(y_{i}-{ŷ}_{i}^{\left(-i\right)}\right)}^{2}$ where ${ŷ}_{i}^{\left(-i\right)}$ estimates the i^th^ response from a model that was estimated without this observation (36). Further, the Akaike Information Criterion (AIC) of each model was compared with the result of the PRESS statistic.

Based on the final regression model, the formula for the demographically corrected standard values (z-scores) was developed using the z-score formula z = (score–expected score)/residual standard deviation. Cutoff scores were developed based on the z-score-formula for the 10th percentile (z = −1.28) for men and women for each age (60–97 years) and all years of education (7–18). Statistics were calculated by the statistical program SPSS (Version 28) and normative data were calculated by Rstudio (2021.09.1). Confidence interval was set at 95% and statistical significance was defined as a *p* < 0.05.

The study was registered on the MoCA homepage (www.mocatest.org). The study met the guidelines of the Declaration of Helsinki, and all participants gave their written consent. All examiners underwent training as required by www.mocatest.org.

## Results

### Demographics

One hundred seventy-five participants aged 60 to 97 years (M = 71.52; SD = 8.77) were included in the present study. 100 subjects were aged between 60 and 71 years (males *n* = 59, females *n* = 41), 60 subjects between 72 and 83 years (males *n* = 33, females *n* = 27) and 15 subjects were aged 84 years or older (males *n* = 7, females *n* = 8). According to the WHO definition, 52 patients did not suffer from hearing loss (WHO 0, 4PTA: 15.45 (SD 5.11) dB), 42 participants were classified as WHO 1 with a mean 4PTA of 30.63 (SD 3.43) dB, 41 were suffering from a hearing loss of 45.70 (SD 5.66) dB in mean (WHO 2). 40 subjects with a mean 4PTA of 88.75 (SD 22.05) belonged to WHO 3 and 4.

**Figure 1:**
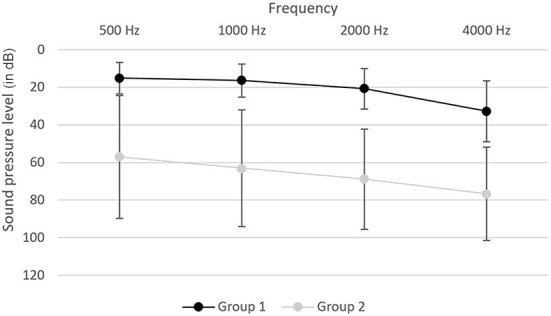
Audiogram of group 1 and group 2. Testing was performed in the frequencies of 500, 1000, 2000 and 4000 Hz and graphs are shown for the mean sound pressure level in dB with the standard deviation on the better hearing ear.

Study samples were divided into 2 groups. Group 1 (94 subjects) was normal hearing or only slightly hearing-impaired (4PTA ≤ 40 dB, WHO 0 and WHO 1) with a mean 4PTA of 22.23 dB (SD 8.78) and group 2 (81 subjects) was moderate or profound hearing-impaired (4PTA > 40 dB, WHO 2, 3 and 4) with a mean 4PTA of 66.96 dB (SD 26.87). Audiometric data are shown in Figure 1. Group 2 [mean age 73.95 (SD 9.22)] was older than group 1 [mean age 69.43 (SD 7.81) (*p* < 0.001)] and had a lower educational level than group 1 (*p* < 0.001) (Table 1).

**Table 1:** Demographic data.

	**Group 1**	**Group 2**
	***M (SD)***	***M (SD)***
Total number of participants	94	81
Total number of male participants	56	43
Total number of female participants	38	38
Age (years)	69.43 (7.81)	73.95 (9.22)
Education (years)	14.07 (3.38)	12.43 (2.93)
4PTA (dB)	22.23 (8.78)	66.96 (26.87)

Group 1 includes normal hearing and mild hearing-impaired participants (4PTA on the better hearing ear of ≤ 40 dB), group 2 includes participants with a moderate to profound hearing impairment (4PTA on the better hearing ear > 40 dB).

### MoCA-HI score and impact of age, sex, education and hearing level

Mean scores of the MoCA-HI and the subdomains in group 1 and group 2 are shown in Table 2A. There was no significant difference between the MoCA-HI total score of group 1 and group 2 (MD = −1.05; *p* = 0.05). However, Welch test showed that group 1 performed significantly better than group 2 in the cognitive subdomain of visuospatial and executive functions (MD = −0.39; *p* = 0.02). None of the other cognitive subdomains, such as naming (MD = −0.03; *p* = 0.36), attention (MD = −0.18; *p* = 0.2), language (MD = −0.12; *p* = 0.33), abstraction (MD = −0.12; *p* = 0.2), and recall (MD = −0.16; *p* = 0.54), showed a significant difference between the two groups in the independent sample *t*-test. For the cognitive subdomain of orientation, Welch's test also showed no significant group difference (MD = −0.04; *p* = 0.28).

**Table 2A:** Mean score in MoCA-HI and the different cognitive subdomains in Group 1 and Group 2.

	**Group 1**	**Group 2**	**Maximum score**
	**N = 94**	**N = 81**	
	***M (SD)***	***M (SD)***	
MoCA-HI (total score)	24.71 (3.51)	23.67 (3.51)	30
Visuospatial/Executive	3.88 (0.98)	3.49 (1.15)	5
Naming	2.97 (0.18)	2.94 (0.24)	3
Attention	5.20 (0.97)	5.02 (0.84)	6
Language	2.27 (0.79)	2.15 (0.79)	3
Abstraction	1.46 (0.67)	1.33 (0.61)	2
Delayed recall	2.98 (1.78)	2.81 (1.78)	5
Orientation	5.96 (0.20)	5.91 (0.32)	6

Group 1 includes normal hearing and mild hearing-impaired participants (4PTA on the better hearing ear ≤ 40 dB), group 2 includes participants with a moderate to profound hearing impairment (4PTA on the better hearing ear > 40 dB). MoCA-HI, Montreal Cognitive Assessment - Hearing impairment.

A multiple linear regression analysis revealed that older age (β = −0.28; *p* < 0.001), male sex (β = −0.16; *p* = 0.009), and lower education (β = 0.48; *p* < 0.001) were associated with a lower MoCA-HI total score and together explained 41.7% (adjusted R^2^ = 0.41) of the total variance (F = 40.73; *p* < 0.001). Mean total scores in the MoCA-HI in the 4 different WHO groups are shown in Table 2B. There was no difference in MoCA-HI performance using the 4PTA as a continuous variable (β = −0.02; *p* = 0.72). Further no significant effect of the level of hearing impairment on the different subscores was found (β ≤ 0.06; *p* ≥ 0.46).

**Table 2B:** Mean values of the MoCA-HI total score, age, education and 4PTA according to the WHO classification.

	**WHO 0**	**WHO 1**	**WHO 2**	**WHO 3 & 4**
	***N* = 52**	***N* = 42**	***N* = 41**	***N* = 40**
	***M (SD)***	***M (SD)***	***M (SD)***	***M (SD)***
MoCA-HI total score	25.60 (3.30)	23.62 (3.49)	23.20 (3.27)	24.15 (3.73)
Age (years)	67.50 (6.10)	71.81 (9.04)	76.37 (9.36)	71.48 (8.50)
Education (years)	14.54 (3.44)	13.50 (3.24)	12.20 (2.88)	12.68 (2.99)
4PTA (dB)	15.45 (5.11)	30.63 (3.43)	45.70 (5.66)	88.75 (22.05)

Normal hearing group, 4PTA on the better hearing ear < 26 dB (WHO 0); WHO 1, 4PTA on the better hearing ear 26–40 dB; WHO 2, 4PTA on the better hearing ear 41-60 dB; WHO 3 & 4, 4PTA on the better hearing ear >60 dB; MoCA-HI, Montreal Cognitive Assessment - Hearing impairment.

A retest was performed in 115 participants. The mean retest interval was 60.38 (SD 18.08) days after the first administration with a minimum of 28 and a maximum of 112 days. Test-retest reliability was high with a Pearson correlation of 0.84 (*p* < 0.001). However, the MoCA-HI total score was higher in the retest than at baseline (MD = 0.44; *p* = 0.008), due to a statistically significant improvement in the cognitive subdomain “recall” in the retest (*p* < 0.001). All other subtests remained stable after re-testing.

### Comparison of the standard MoCA with the MoCA-HI

There was no difference between group 1 (mean rank = 89.70) and group 2 (mean rank = 86.03) in the adapted items of the MoCA-HI (*p* = 0.58). In contrast, individuals of group 1 (mean rank = 106.27) performed significantly better than subjects of group 2 (mean rank = 66.80) on the sum of the corresponding items of the standard MoCA (*p* < 0.001). Further, group 1 did not differ in the MoCA and MoCA-HI performance (*p* = 0.12), whereas group 2 performed significantly better on the MoCA-HI than on the standard MoCA (*p* < 0.001).

### Establishment of normative data

**Figure 2:**
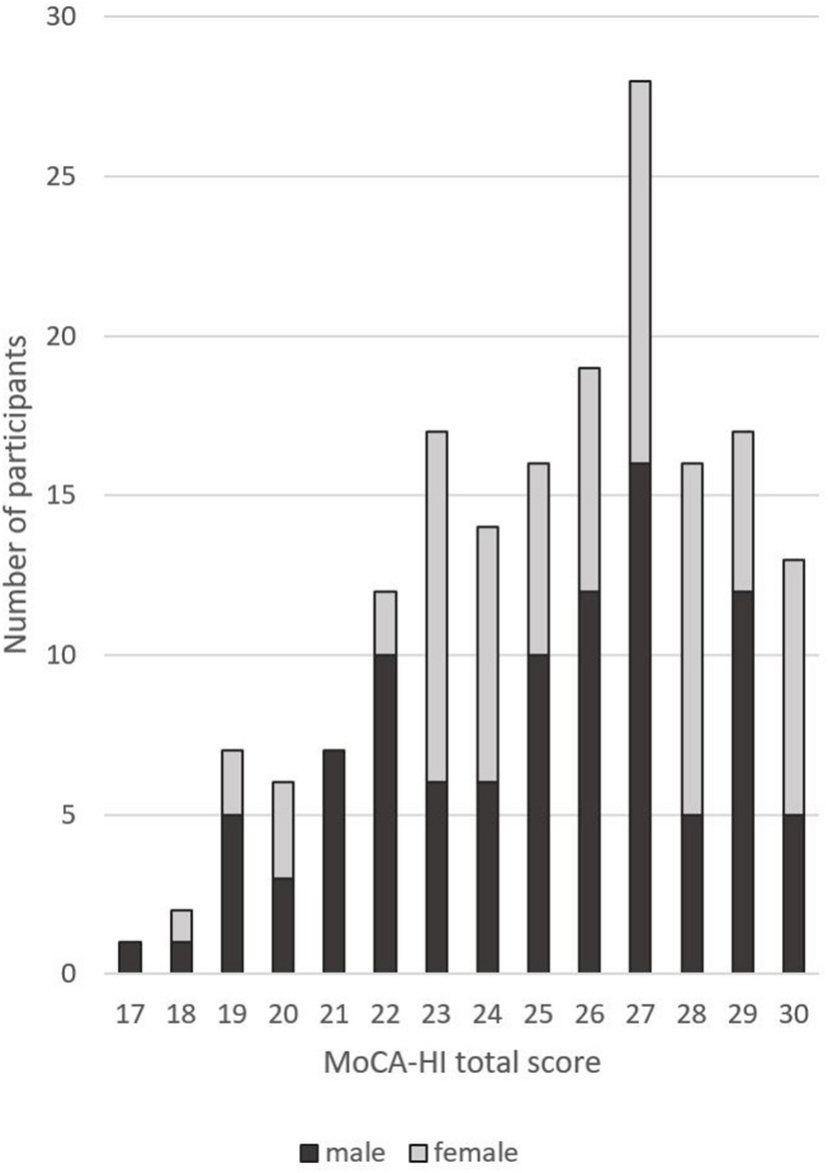
Distribution of the adjusted MoCA-HI total score.

**Figure 3:**
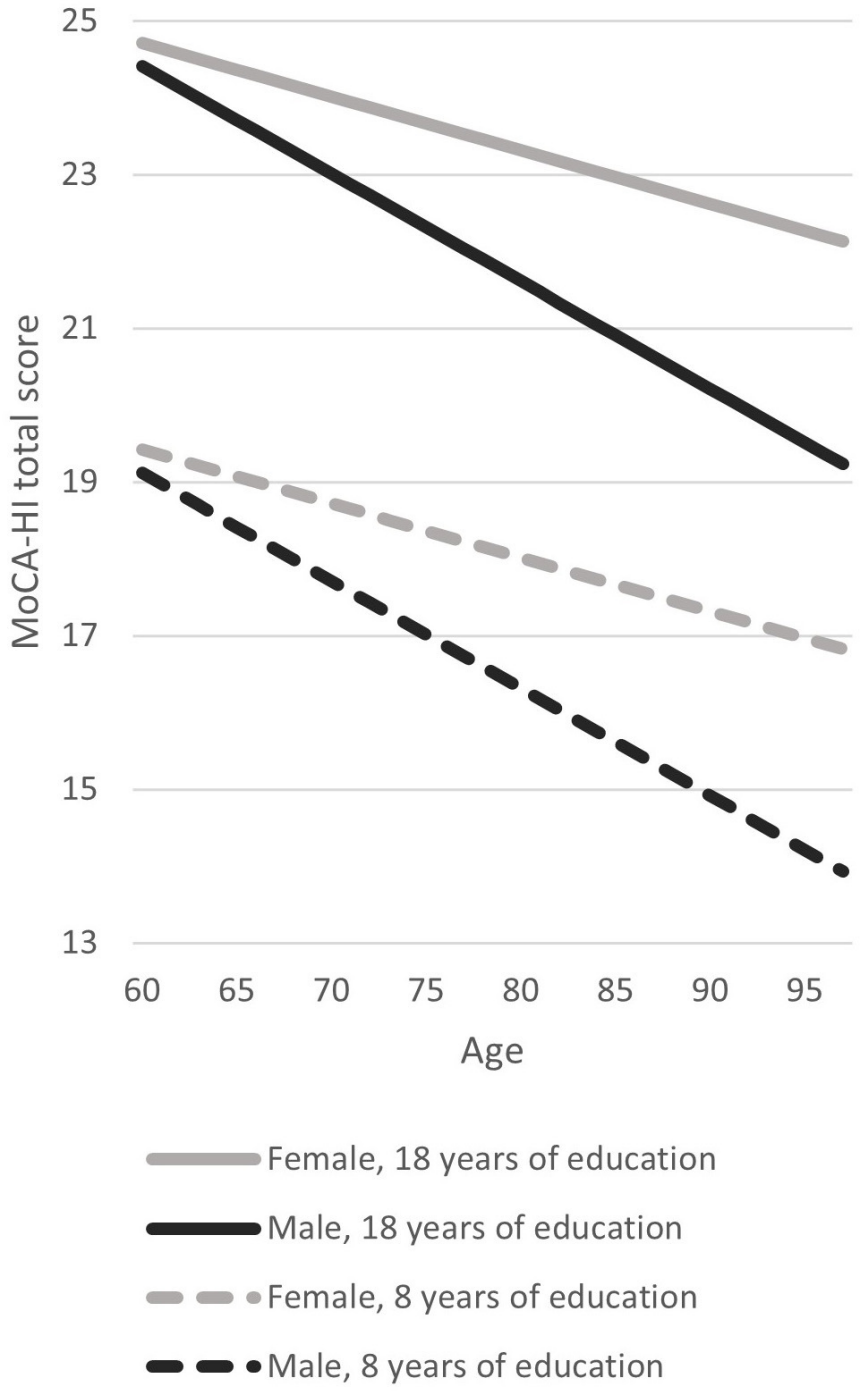
Example regression lines for the whole study sample (*n* = 175) representing the relationship of MoCA-HI total score with age, education and sex. Example regression lines are shown for subjects with 8 and 18 years of education. The regression model shows that the MoCA-HI total score was lower in case of less educational years, an increasing age and in male sex. Age had a stronger effect on the MoCA-HI total score in men than in women. 100 of the subjects included were aged between 60 and 71 years (males *n* = 59, females *n* = 41), 60 subjects between 72 and 83 years (males *n* = 33, females *n* = 27) and 15 subjects were aged 84 years or older (males *n* = 7, females *n* = 8).

In a first step, MoCA-HI scores were adjusted for education as suggested by Dawes (www.mocatest.org), showing that 35.5% of women and 39.4% of men scored below the original cutoff (see Figure 2). Therefore, in a second step normative data for the German version of the MoCA-HI were calculated taking into account education as well as age and sex using a regression-based approach (Figure 3). A regression model including age, years of education, sex and the interaction of age and sex as covariates had both the lowest PRESS statistic and the lowest AIC and was thus the best predicting model for the MoCA-HI total score, which explained 42.35% of the variance (adjusted R^2^ = 0.41; F = 31.22; *p* < 0.001). This effect is strongest for education (t = 7.52), followed by sex (t = −2.65), age (t = −1.86), and the interaction of age and sex (t = −1.41), as indicated by the *t*-values. Based on the present data, the z-Score could be determined as follows: z = (Score–(22.86 + (−0.07 ^*^ age) + (0.53 ^*^ education) + (−1.11 ^*^ sex) + (−0.07 ^*^ (age−71.52) ^*^ sex)))/2.72. Sex was coded as 0 = female and 1 = male, age and education are inserted in years. The resulting cutoff scores for the 10th percentile are shown in Tables 3A,B.

**Table 3A:** Highest MoCA-HI total scores just below the 10th percentile for women (z-score ≤ −1.28).

		**Education (in years)**											
**Age (in years)**		7	8	9	10	11	12	13	14	15	16	17	18
	60	19	19	20	20	21	21	22	23	23	24	24	25
	61	19	19	20	20	21	21	22	22	23	24	24	25
	62	19	19	20	20	21	21	22	22	23	23	24	25
	63	19	19	20	20	21	21	22	22	23	23	24	24
	64	19	19	20	20	21	21	22	22	23	23	24	24
	65	18	19	20	20	21	21	22	22	23	23	24	24
	66	18	19	19	20	21	21	22	22	23	23	24	24
	67	18	19	19	20	20	21	22	22	23	23	24	24
	68	18	19	19	20	20	21	21	22	22	23	24	24
	69	18	19	19	20	20	21	21	22	22	23	23	24
	70	18	19	19	20	20	21	21	22	22	23	23	24
	71	18	19	19	20	20	21	21	22	22	23	23	24
	72	18	18	19	20	20	21	21	22	22	23	23	24
	73	18	18	19	19	20	21	21	22	22	23	23	24
	74	18	18	19	19	20	20	21	22	22	23	23	24
	75	18	18	19	19	20	20	21	21	22	23	23	24
	76	18	18	19	19	20	20	21	21	22	22	23	24
	77	18	18	19	19	20	20	21	21	22	22	23	23
	78	18	18	19	19	20	20	21	21	22	22	23	23
	79	17	18	19	19	20	20	21	21	22	22	23	23
	80	17	18	18	19	20	20	21	21	22	22	23	23
	81	17	18	18	19	19	20	21	21	22	22	23	23
	82	17	18	18	19	19	20	20	21	22	22	23	23
	83	17	18	18	19	19	20	20	21	21	22	22	23
	84	17	18	18	19	19	20	20	21	21	22	22	23
	85	17	18	18	19	19	20	20	21	21	22	22	23
	86	17	17	18	19	19	20	20	21	21	22	22	23
	87	17	17	18	18	19	20	20	21	21	22	22	23
	88	17	17	18	18	19	19	20	21	21	22	22	23
	89	17	17	18	18	19	19	20	20	21	22	22	23
	90	17	17	18	18	19	19	20	20	21	21	22	23
	91	17	17	18	18	19	19	20	20	21	21	22	22
	92	17	17	18	18	19	19	20	20	21	21	22	22
	93	16	17	18	18	19	19	20	20	21	21	22	22
	94	16	17	17	18	19	19	20	20	21	21	22	22
	95	16	17	17	18	18	19	20	20	21	21	22	22
	96	16	17	17	18	18	19	19	20	21	21	22	22
	97	16	17	17	18	18	19	19	20	20	21	21	22

Odd numbers are highlighted in white and even numbers in gray.

**Table 3B:** Highest MoCA-HI total scores just below the 10th percentile for men (z-score ≤ -1.28).

		**Education (in years)**											
**Age (in years)**		7	8	9	10	11	12	13	14	15	16	17	18
	60	18	19	20	20	21	21	22	22	23	23	24	24
	61	18	19	19	20	20	21	22	22	23	23	24	24
	62	18	19	19	20	20	21	21	22	22	23	24	24
	63	18	19	19	20	20	21	21	22	22	23	23	24
	64	18	18	19	20	20	21	21	22	22	23	23	24
	65	18	18	19	19	20	20	21	22	22	23	23	24
	66	18	18	19	19	20	20	21	21	22	22	23	23
	67	18	18	19	19	20	20	21	21	22	22	23	23
	68	17	18	18	19	20	20	21	21	22	22	23	23
	69	17	18	18	19	19	20	20	21	21	22	23	23
	70	17	18	18	19	19	20	20	21	21	22	22	23
	71	17	17	18	19	19	20	20	21	21	22	22	23
	72	17	17	18	18	19	19	20	21	21	22	22	23
	73	17	17	18	18	19	19	20	20	21	21	22	23
	74	17	17	18	18	19	19	20	20	21	21	22	22
	75	16	17	17	18	19	19	20	20	21	21	22	22
	76	16	17	17	18	18	19	19	20	21	21	22	22
	77	16	17	17	18	18	19	19	20	20	21	21	22
	78	16	17	17	18	18	19	19	20	20	21	21	22
	79	16	16	17	17	18	19	19	20	20	21	21	22
	80	16	16	17	17	18	18	19	19	20	20	21	22
	81	16	16	17	17	18	18	19	19	20	20	21	21
	82	15	16	16	17	18	18	19	19	20	20	21	21
	83	15	16	16	17	17	18	18	19	20	20	21	21
	84	15	16	16	17	17	18	18	19	19	20	20	21
	85	15	16	16	17	17	18	18	19	19	20	20	21
	86	15	15	16	16	17	18	18	19	19	20	20	21
	87	15	15	16	16	17	17	18	18	19	20	20	21
	88	15	15	16	16	17	17	18	18	19	19	20	20
	89	14	15	16	16	17	17	18	18	19	19	20	20
	90	14	15	15	16	16	17	18	18	19	19	20	20
	91	14	15	15	16	16	17	17	18	18	19	19	20
	92	14	15	15	16	16	17	17	18	18	19	19	20
	93	14	14	15	16	16	17	17	18	18	19	19	20
	94	14	14	15	15	16	16	17	17	18	19	19	20
	95	14	14	15	15	16	16	17	17	18	18	19	19
	96	13	14	15	15	16	16	17	17	18	18	19	19
	97	13	14	14	15	15	16	17	17	18	18	19	19

Odd numbers are highlighted in white and even numbers in gray.

## Discussion

This present study is the first to evaluate the German MoCA-HI in normal-hearing and hearing-impaired subjects and to develop normative data for cognitively healthy individuals adjusted for age, education and sex.

### Development of a MoCA version for hearing-impaired

There have been two previous attempts to adapt the MoCA for people with hearing loss. Dupuis et al. adapted the standard MoCA by removing spoken items (sentence repetition, lists of numbers, list of letters, delayed recall) from the assessment and established new cutoff scores proportionally adjusted for the deleted items (18). However, Al-Yawer found in a retrospective analysis that this approach reduced the sensitivity of the test scores of patients with mild cognitive impairment from 90 to 56%, although sensitivity for dementia was not affected (23).

Lin et al. developed a timed computerized visual version of the MoCA and reported no difference in performance of the computerized visual MoCA between cognitively normal participants with normal hearing (*n* = 103) vs. hearing loss (*n* = 49) (37). Lerch and Benz created a German language version of Lin et al.'s computerized MoCA and tested it in 50 normal hearing and 100 hearing-impaired participants (38). A comparison with the Consortium to Establish a Registry for Alzheimer's Disease (CERAD) and the Mini Mental Status Examination (MMSE) showed that the computerized MoCA-HI correlated with the CERAD plus battery (38). Utoomprurkporn et al. (2021) tested a modified version of Lin et al.'s computerized visual MoCA in 75 hearing aid users (39), 30 cognitive healthy, 30 with MCI and 15 with a clinical diagnosis of dementia reporting good sensitivity and specificity for MCI and dementia in their analysis. However, the small sample size and group differences in age and educational level limit the reliability of sensitivity/specificity estimates.

The visual version of the MoCA reported in the current study has several advantages over previous versions of the MoCA adapted for people with hearing loss. First, rather than deleting spoken items, it replaces the standard spoken items by other items that tap into the same cognitive domain and are of a similar level of difficulty. Secondly, it was validated in a large cohort in a multi-centre study (25, 26). Thirdly, it may be administered in either paper-and-pencil format or computerized presentation.

### Performance of the German version of the MoCA-HI and the MoCA in participants with vs. without hearing loss

Group 2 (hearing loss of ≥ 41 dB) performed worse than group 1, which had no hearing loss or only a mild hearing loss, on the standard MoCA, but not on the three adapted tasks of the MoCA-HI. In line with that, there was no significant difference in the performance in the MoCA and MoCA-HI of group 1, while group 2 performed significantly worse in the standard MoCA than in the MoCA-HI. Thus, at least people with a severe hearing impairment may benefit from a visual version of the MoCA and the MoCA-HI may prevent false-positive diagnosis of dementia especially in case of a severe or profound hearing loss (19). However, the impact of a mild hearing loss cannot be answered right now and should be studied in larger samples in the future.

### Impact of age, education, sex and level of hearing loss on performance

The MoCA-HI total score was best predicted by a regression model including age, education, sex, and the interaction of age and sex; age had a stronger effect on the total score in men than in women. This is in line with previous studies on the original MoCA, where regression models including age, education and sex had the best predictive power (33, 35, 40, 41). Given these differences, age-, education- and sex-specific normative values were developed to adjust for these demographic variables and to optimize the detection of cognitive impairment for the German MoCA-HI version. Hearing status based on 4PTA of the better ear did not impact on the total MoCA-HI performance as shown by regression analysis taking into account age, sex and educational level. Therefore, even if the WHO classification of the 4PTA of the better hearing ear used in the present study does not fully reflect the hearing abilities in daily life, this cognitive test battery seems to be suitable for anyone regardless of the hearing level.

### Re-test reliability

To use the MoCA-HI in clinical practice, a re-test is necessary. In the present study participants performed slightly better in the re-test with less than 1 point more. Although this improvement was statistically significant, it did not make a difference to the clinical classification on the MoCA-HI. Practice effects cannot fully be ruled out in re-testing (42), although Faletti et al., have demonstrated that an interval of 4 weeks between testing and re-testing might be sufficient (43). In the present study the better performance in the re-test was only due to the large improvement in the recall subtest. Therefore, a further version of the MoCA-HI should be developed, including new terms in the MoCA subtest recall, before introducing the MoCA-HI assessment into clinical routine.

### Limitations

One limitation of our study is that we relied on the GPCOG to establish normal cognition criteria. The GPCOG is somewhat like the standard MoCA in including spoken items, so hearing status may have impacted categorization as normal cognition based on GPCOG performance. Some people with hearing loss might have been incorrectly excluded. However, we do not consider this to be a serious issue, since it was our aim to include only cognitive healthy individuals in this analysis.

Previous research indicated that performance criteria to identify cognitive impairment developed in English may not be applicable to translations of the MoCA in other languages (27). Cut-points for the English MoCA-HI may not be applicable to the German MoCA-HI. In a follow-up project, we are currently collecting data to establish optimal performance criteria for identification of cognitive impairment for the German MoCA-HI. The analysis of demographic correlates of performance reported in the current paper suggests that adjustments for age, sex and educational level may facilitate optimal discriminative power.

## Conclusion

The German translation of the MoCA-HI is suitable in subjects with and without hearing loss and has high retest reliability. Performance criterion for identification of cognitive impairment should be developed, considering the impact of age, sex and educational level. A language-specific validation is required due to linguistic and cultural differences.

## Data availability statement

The original contributions presented in the study are included in the article/supplementary material, further inquiries can be directed to the corresponding author.

## Ethics statement

The present study was approved by the Ethic Institution of the Ruhr-University Bochum (No. 20-7076). The patients/participants provided their written informed consent to participate in this study.

## Author contributions

CV, LG, and PD designed the study. HF collected the data and did the statistical analysis with critical feedback from FL and MT. CV and HF wrote the manuscript with contributions from PD and critical feedback from LG, ZN, and RW. All authors contributed to the article and approved the submitted version.
